# Bait Preference of Free-Ranging Feral Swine for Delivery of a Novel Toxicant

**DOI:** 10.1371/journal.pone.0146712

**Published:** 2016-01-26

**Authors:** Nathan P. Snow, Joseph M. Halseth, Michael J. Lavelle, Thomas E. Hanson, Chad R. Blass, Justin A. Foster, Simon T. Humphrys, Linton D. Staples, David G. Hewitt, Kurt C. VerCauteren

**Affiliations:** 1 Caesar Kleberg Wildlife Research Institute, Texas A&M University-Kingsville, 700 University Blvd., MSC 218, Kingsville, Texas, 78363, United States of America; 2 USDA/APHIS/ Wildlife Services, National Wildlife Research Center, 4101 LaPorte Ave., Fort Collins, Colorado, 80521, United States of America; 3 Kerr Wildlife Management Area, Texas Parks and Wildlife Department, 2625 FM 1340, Hunt, Texas, 78024, United States of America; 4 Invasive Animals CRC, 33 Flemington St., Glenside, South Australia, 5062, Australia; 5 Animal Control Technologies Australia Pty Ltd, 46–50 Freight Dr., Somerton, Victoria, 3062, Australia; US Geological Survey, UNITED STATES

## Abstract

Invasive feral swine (*Sus scrofa*) cause extensive damage to agricultural and wildlife resources throughout the United States. Development of sodium nitrite as a new, orally delivered toxicant is underway to provide an additional tool to curtail growth and expansion of feral swine populations. A micro-encapsulation coating around sodium nitrite is used to minimize detection by feral swine and maximize stability for the reactive molecule. To maximize uptake of this toxicant by feral swine, development a bait matrix is needed to 1) protect the micro-encapsulation coating so that sodium nitrite remains undetectable to feral swine, 2) achieve a high degree of acceptance by feral swine, and 3) be minimally appealing to non-target species. With these purposes, a field evaluation at 88 sites in south-central Texas was conducted using remote cameras to evaluate preferences by feral swine for several oil-based bait matrices including uncolored peanut paste, black-colored peanut paste, and peanut-based slurry mixed onto whole-kernel corn. These placebo baits were compared to a reference food, whole-kernel corn, known to be readily taken by feral swine (i.e., control). The amount of bait consumed by feral swine was also estimated using remote cameras and grid boards at 5 additional sites. On initial exposure, feral swine showed reduced visitations to the uncolored peanut paste and peanut slurry treatments. This reduced visitation subsided by the end of the treatment period, suggesting that feral swine needed time to accept these bait types. The black-colored peanut paste was visited equally to the control throughout the study, and enough of this matrix was consumed to deliver lethal doses of micro-encapsulated sodium nitrite to most feral swine during 1–2 feeding events. None of the treatment matrices reduced visitations by nontarget species, but feral swine dominated visitations for all matrices. It was concluded that black-colored peanut paste achieved satisfactory preference and consumption by feral swine, and no discernable preference by non-target species, compared to the other treatments.

## Introduction

Feral swine (*Sus scrofa*) are an invasive species in the United States that have dramatically increased in both abundance and geographic range in the last 30 years [1]. This increase can be attributed to adaptability, high reproductive potential [2], lack of predators [1], intentional and accidental introductions by humans [3] and their adaptability to occupy a variety of landscapes and opportunistically feed on many food items. Since 2000, as many as 47 of the 50 United States have reported observations of feral swine, 36 of which have established populations [4]. Consequently, feral swine are the most abundant introduced ungulate in the United States [5].

The rapid expansion of feral swine populations has increased costs associated with damage they cause and control efforts [6, 7]. Economic losses estimated at $1.5 billion are incurred each year in the United States due to losses from crop damage, depredation of livestock, spread of disease, and the cost of labor and equipment for control efforts [7]. Additionally, feral swine harm native ecosystems and natural resources by reducing plant species diversity [8], depredating sensitive species [9, 10], and destroying habitat and nests of desired native species [11]. Previous studies have demonstrated that current control strategies for feral swine can generate local reductions in populations (e.g., [2, 12, 13–15]). These strategies include trapping, snaring, recreational hunting, professional sharpshooting, and aerial shooting. However, it is apparent that these methods are insufficient, given the increase in abundance and distribution of feral swine, and subsequent rise in human-related conflicts [16, 17]. Therefore, to reduce the impacts of feral swine, additional new tools are needed to help control existing populations and curtail the expansion of range [5].

One new tool with potential to be broadly applied in a cost-effective manner is a humane-and fast-acting toxicant for feral swine [18–21]. Currently, there are no registered toxicants for use on feral swine in the United States [18]. An international team including the National Wildlife Research Center (the research branch of the USDA/APHIS/Wildlife Services), USA; the Invasive Animal Cooperative Research Center, University of Canberra, AU; Animal Control Technologies Australia Pty Ltd, AU; and the Texas Parks and Wildlife Department, USA has been conducting research to develop a toxicant that is humane, cost-effective, and species targeted for use on feral swine in the United States. The proposed end-use product is a manufactured bait or bait concentrate that conceals a 10% concentration of the active ingredient, sodium nitrite. A micro-encapsulation coating is used to conceal and maximize stability of sodium nitrite, while also leaving it bioavailable once consumed. In addition, a bait matrix that is oil-based helps maintain stability of sodium nitrite by providing a temporary hydrophobic barrier, and helps achieve a homogeneous mixing with the micro-encapsulated active ingredient. Sodium nitrite humanely and quickly kills feral swine by methemoglobinemia when a lethal dose is consumed [22]. Methemoglobinemia has also been reported in other species [23], therefore concern for nontarget animals exists.

Attraction of feral swine to an oil-based matrix is unknown. Previous studies have shown that selecting suitable matrices for feral swine is difficult [19, 20]. Novel food items generate neophobic behaviors by domestic swine [24], and may inadvertently provide nontarget species with an attractive food source [19, 20, 25]. These responses represent major challenges for delivering bait to feral swine. Our objective was to evaluate preference of free-ranging feral swine for 3 oil-based matrices, and compare these to a reference food item that is known to be highly attractive to feral swine, whole kernel corn [26]. Secondly, we evaluated preferences of nontarget species to minimize their attraction to the matrices. Thirdly, we estimated the amount of matrices consumed by individual feral swine to evaluate whether enough was consumed to deliver a lethal dose of sodium nitrite. Results from this study provide information to support the possible registration of a toxicant for feral swine through the United States Environmental Protection Agency.

## Materials and Methods

### Study Area

**Fig 1:**
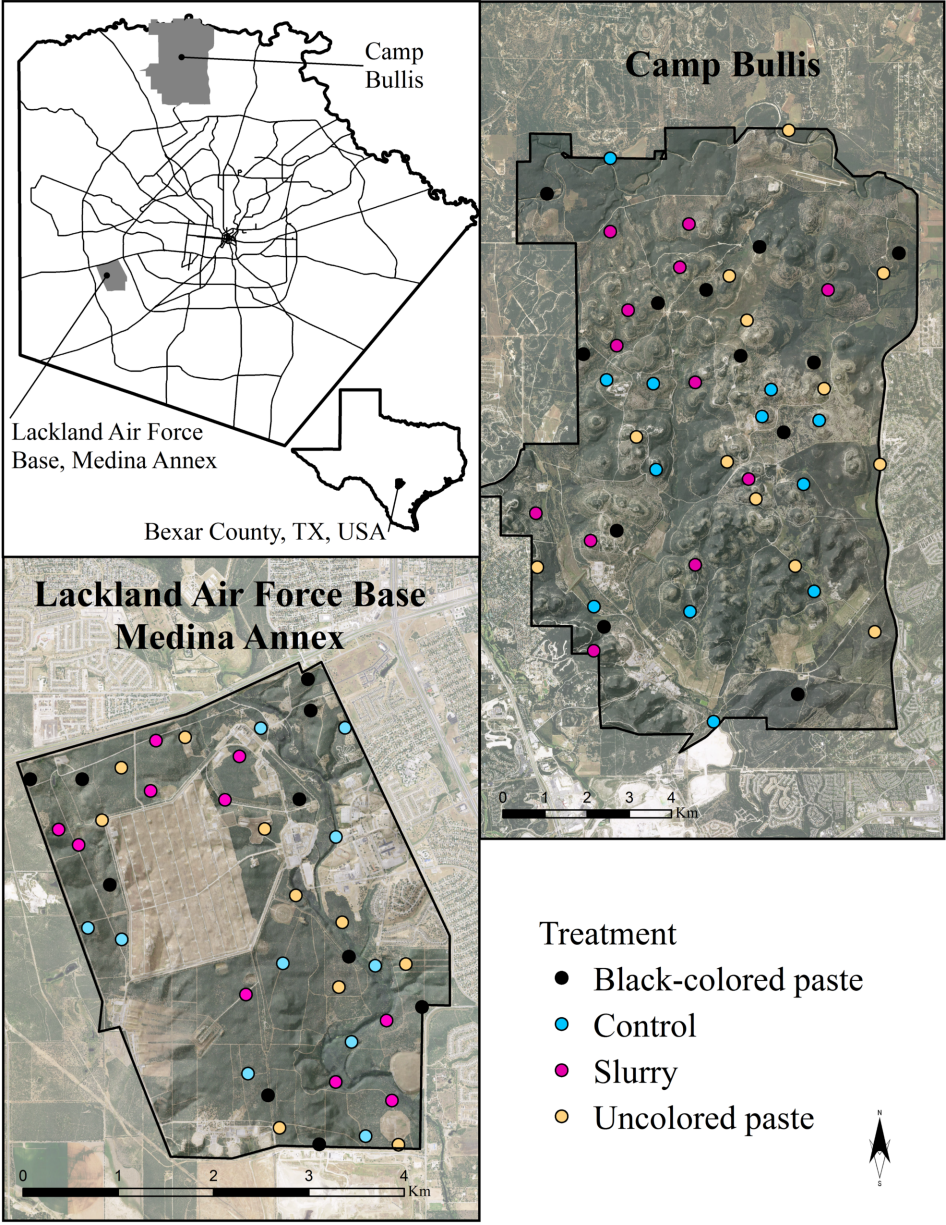
Study area and *n* = 88 randomly generated sample points on Camp Bullis and Lackland Air Force Base Medina Annex, Bexar County, TX, USA during April–May 2015. Background imagery is from the 2014 USDA, Farm Service Agency, National Agriculture Imagery Program (NAIP).

**Fig 2:**
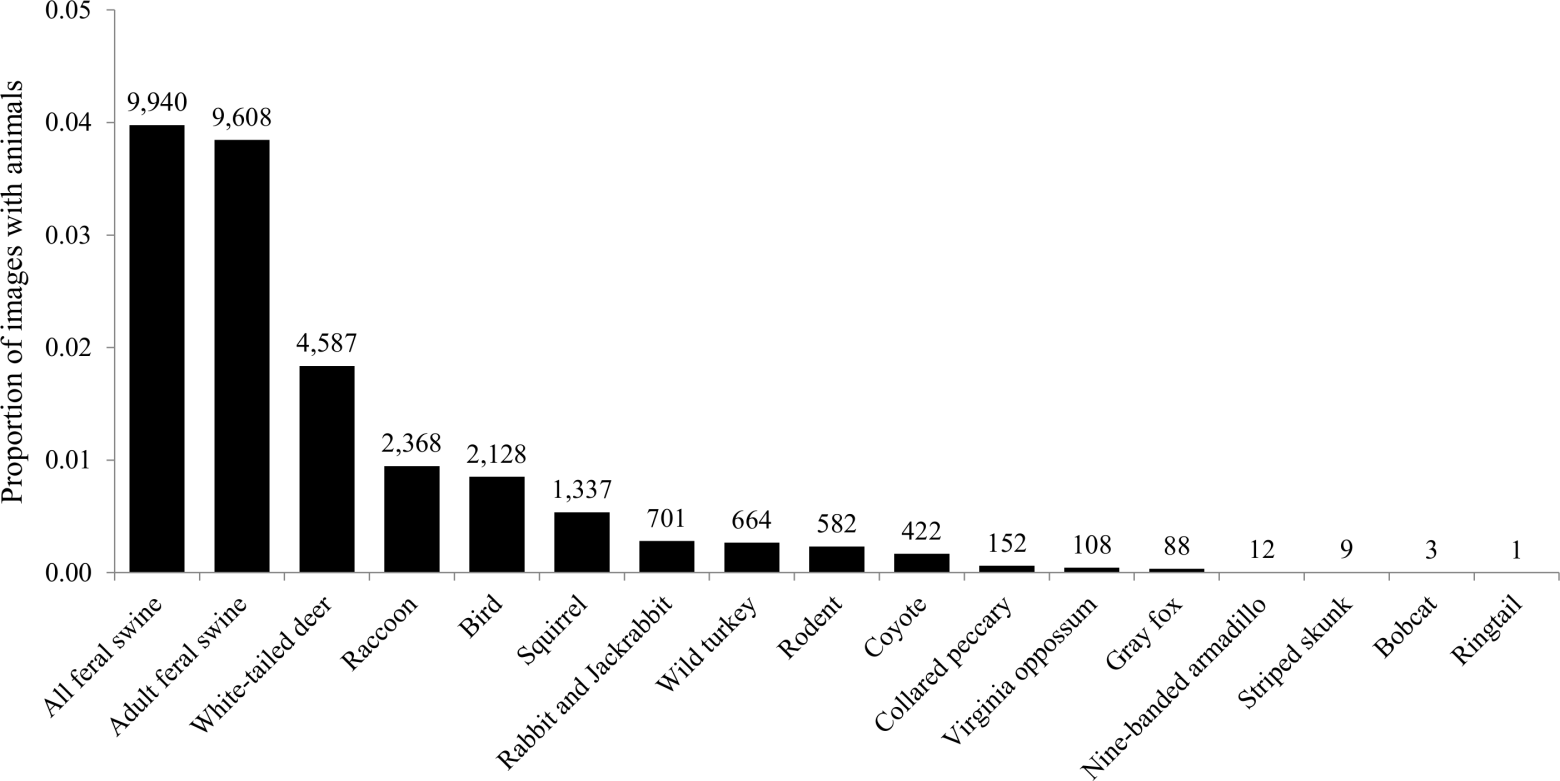
The proportion of 249,902 total images with animals from 87 random bait sites on Camp Bullis and Lackland Air Force Base Medina Annex, TX, USA during April–May 2015. Identified species include feral swine (*Sus scrofa*), white-tailed deer (*Odocoileus virginianus*), raccoons (*Procyon lotor*), passerine and scavenger birds, squirrels (*Sciurus* spp.), cottontail rabbits (*Sylvilagus* spp.) and black-tailed jackrabbits. (*Lepus californicus*), wild turkeys (*Meleagris gallopavo*), rodents, coyotes (*Canis latrans*), collared peccary (*Pecari tajacu*), Virginia opossums (*Didelphis virginiana*), gray foxes (*Urocyon cinereoargenteus*), nine-banded armadillos (*Dasypus novemcinctus*), striped skunks (*Mephitis mephitis*), bobcats (*Lynx rufus*), and a ringtail (*Bassariscus astutus*). Numbers above bars represent the counts of images.

All experimental activities were conducted on two military properties (Fig 1) operated by Joint Base San Antonio, Texas, USA, Camp Bullis (112.9 km^2^) and Lackland Air Force Base Medina Annex (16.1 km^2^). Permission for conducting research on these properties was provided by the Natural Resources office of Joint Base San Antonio, Camp Bullis. These properties reside in the Edwards Plateau and Blackland Prairie ecoregions of the south-central semi-arid prairies of Texas, respectively [27, 28]. Average temperature during the study varied from 9.4–32.8°C (in April and May 2015; National Climatic Data Center). Average daily precipitation was 6.4 mm in April and 7.1 mm in May 2015. Population control of feral swine had not been administered for 3–5 years prior to study, with the exception of limited recreational hunting on Camp Bullis. Both properties were restricted access and surrounded by perimeter fencing. A list of observed nontarget species for the study areas is provided in Fig 2.

### Candidate Bait Matrix Treatments

Three formulations of bait matrices manufactured by Animal Control Technologies P/L (Somerton, Victoria, AU) were tested. The first matrix was uncolored peanut paste mixed with crushed corn and wheat. The second matrix was a black-colored formulation identical to the first except for the addition of ~0.1% ferric oxide as an odorless and tasteless black colorant. The black colorant was added in an attempt to reduce visual appeal of the paste to children and wildlife, especially birds that tend to prefer red and yellow food items [29, 30–32]. The third matrix was a slurry of thickened peanut oil and peanut paste that could be poured over a carrier of choice, in this case whole-kernel corn. We compared these 3 treatment matrices to a reference bait of plain whole-kernel corn (i.e., control bait). Whole-kernel corn provides a good reference because it is commonly used to attract feral swine, and has been shown to keep feral swine at bait sites [26].

### Study Design for Bait Preference

Baiting sites were located using a Geographic Information System (ArcGIS v10.2, ESRI Redlands, CA) to identify and select land covers that represented areas preferred by feral swine. Specifically, we used the 2006 National Land Cover Database [33, 34] to identify areas with trees and shrubs (deciduous and coniferous), as well as grass-dominated land covers. We excluded land covers that represented developed, open water, and barren areas. Exclusion areas were also created 300 m from buildings and 100 m from roads, core areas of endangered golden-cheeked warblers (*Setophaga chrysoparia*), karsts and caves, and restricted cultural areas. The remaining areas available for study covered 46.4 km^2^ in Camp Bullis and 8.2 km^2^ in the Lackland Air Force Base Medina Annex.

We generated sets of 48 and 40 spatially balanced and random sites using the Spatially Balanced Points tool in AcrGIS [35] for Camp Bullis and Lackland Air Force Base Medina Annex, respectively. All points were selected to be a minimum of 300 m apart, but averaged 725.1 m apart (SD = 480.0), to reduce potential confounding among points. Each site was randomly assigned to 1 of 4 treatments: 1) uncolored paste, 2) black-colored paste, 3) slurry, or 4) whole-kernel corn, with an equal number of each bait type being assigned to each study area.

We baited and monitored all sites for 10 consecutive days during April 7–17, 2015 on Camp Bullis and April 23–May 3, 2015 on Lackland Air Force Base Medina Annex. We visited each site ≥2 weeks prior to bait deployment to clear vegetation and install a T-post for mounting a remote camera. After this acclimation period, we placed RECONYX PC900 remote cameras (RECONYX, Inc., Holmen, WI, USA) on the T-posts or trees and initiated the baiting study. We mounted cameras facing north 5 m from the bait, 1.5 m above the ground, and an angle of 70° to provide consistent field of view at each site that was approximately 12 m long x 8 m wide. We captured images at time-lapse intervals of 5 min, which has been shown to be sufficient for detecting feral swine at bait sites [26]. We examined the duration of visitation at the bait sites by feral swine by calculating the time between the first image containing feral swine and the last image, with 30 min quiet periods between separate visitations [26].

We deployed 5 kg of bait within a 0.5 m diameter circle at each site. Any uneaten bait was removed and replaced with new bait each day. Days 1–5 were used as a pre-baiting period to allow time for animals to locate sites. Whole-kernel corn was offered at each site during the pre-baiting period. Days 6–10 were the experimental period during which the treatment matrices were offered. For both paste matrices, we progressively increased the ratio of paste to corn throughout the experimental period to provide a gradual transition from corn to paste. One kg of paste and 4 kg of corn were offered during Days 6–8; 3 kg of paste and 2 kg of corn were offered during Day 9; and 5 kg of paste was offered during Day 10. The pastes were flattened into pancake-like forms and placed on top of the corn (if offered). For the slurry matrix, we poured 1 kg (1 L) of the slurry onto 4 kg of whole-kernel corn and thoroughly mixed for Days 6–10, which also represented a soft exposure to the matrix. We continued to offer 5 kg of whole-kernel corn at all control sites during Days 6–10.

At the end of each 10-day treatment period, cameras were removed and all images assessed using the Colorado Parks and Wildlife (CPW) Photo Database (v3.0) for image processing [36]. A blind, double-observer technique was used to identify and count the number of species in each image. The total number of feral swine and the total number of adult feral swine (i.e., estimated >20 kg) were counted. Weight estimation was standardized among observers using example images prior to image processing and knowledge of weights from captive feral swine. We considered all nontarget species separately, with the exception of all birds except for wild turkeys (*Meleagris gallopavo*), combing cottontail rabbits (*Sylvilagus* spp.) and black-tailed jackrabbits (*Lepus californicus*), and all rodents except for squirrels (*Sciurus* spp.).

### Study Design for Bait Consumption

Five additional sites were chosen on Camp Bullis that were ≥400 m from any of the bait preference sites and showed recent sign of feral swine activity (e.g. active wallows). These sites were fitted with 1.2 x 2.4 m sheets of black plywood, overlaid with 20 grid squares of equal size using white paint laid on the ground. A remote camera was mounted above the grid boards following similar methodology described above. Each grid board was pre baited with 5 kg of corn for 2 days. If no feral swine were detected, the grid boards were re-located to new sites.

After the prebaiting period, we placed known quantities of a randomly selected treatment matrix onto each square of the grid board using a standardized scoop. One scoop of the uncolored paste and black-colored paste weighed ~70 g, and 1 scoop of slurry on corn weighed ~62.5 g using the same slurry:corn ratio described above. We monitored consumption of the entire baits from within the grid squares using motion-activated photography that recorded images at 1 second intervals with no delays between activations. We removed uneaten baits and replaced the baits daily for 4–7 days at each site. From the imagery, we identified individual feral swine using the animal’s size, shape, markings and scars to record the amount of bait an animal consumed during a visitation event. In some cases individual animals could not be identified and were excluded from analysis. Independent visitations were considered as feeding events by any animal that occurred ≥2 min after the previous visit. All components of this study were approved by the National Wildlife Research Center Institutional Animal Care and Use Committee (protocol: QA-2439).

### Data Analysis

For analysis of bait preference, we considered 2 possible response variables including: 1) the count of individual animals by species, and 2) the count of images by species, both standardized by the total number of daily images. We considered these metrics separately because of uncertainty in how group feeding behavior may affect inferences. For example, if large groups of animals spend less time at bait sites because they consumed the bait faster than a solitary animal, this could confound inferences if the counts of images are used to indicate preference. However, we used Program R (v3.1.1; R Development Core Team) to examine the Pearson correlation coefficients of these variables and found high correlation (i.e., *r* = 0.88 for adult feral swine and r = 0.69 for all feral swine). This correlation indicated that the 2 variables provided similar information, thus we proceeded with the count of images by species per total daily images as our response variable. This variable was standardized among sites because of our methodology using time-lapse imagery, and therefore provides an index for comparing how the baits affected visits by each species. We used the amount of visitation by each species as a proxy for comparing bait preference.

We examined how the response variable changed through time by dividing the 10 day treatment period into 4 periods. The periods considered were: 1) Days 1–5 as the pre-baiting period, 2) Days 6–10 as the whole treatment period, 3) Days 9–10 as the last 2 days of treatment, and 4) Day 10 as the last day of treatment. We examined histograms and the mean counts of images with animals (and standard errors) for each treatment and day.

Bayesian procedures were used to fit compound Poisson linear mixed models following the Tweedie distribution with package cplm in Program R [37] because both metrics followed continuous distributions with positive values and frequent zeros. Effects from the treatment matrices (fixed effect) grouped by site and by day (random effects) were examined for influence on the response variables. We also examined whether treatment matrices influenced the time (in minutes) it took for bait to be totally consumed. For all analyses, we used control sites as a reference for comparison and Markov chain Monte Carlo methods with 3 chains of 11,000 iterations with random starting values and burn-ins of 1,000 iterations. The chains were examined for convergence using density plots and trace plots. We examined the 95% credible intervals (CI) of the parameter estimates for a lack of overlap on zero to indicate statistical and biological influences from the treatments on the response variable.

*Post hoc*, we examined for differences in temperature between the uncolored and black-colored peanut pastes when exposed to sunlight. We recorded temperatures every hour for 3 flattened spheres (~140 g each) of uncolored and black-colored pastes, respectively, which were exposed to sunlight from 0800–2000 hrs using an infrared thermometer (Traceable™ Infrared Thermometer, Fischer Scientific™, Pittsburgh, PA, USA). We examined if the mean temperature differed among the pastes using Bayesian estimation for two groups (BEST) with package BEST in Program R [38]. We also examined if feral swine visited sites with the black-colored paste more often than uncolored paste during the daylight hours. It was hypothesized that warmer peanut paste could produce more scent that might attract feral swine. We compared the sum of images containing feral swine from 0800–2000 hours at black-colored and uncolored paste sites using BEST.

For analysis of bait consumption, feeding events in which individual feral swine were identified and consumed a measurable amount of bait were considered as the sample units. The response variable was the amount of bait consumed during a feeding event. Influences from the treatment matrices and whether or not other feral swine were eating simultaneously (fixed effects) on the response variable were tested. Both fixed effects were grouped by individuals nested within sites (random effects). We used a similar Bayesian procedure to fit the mixed model as described above; however, black-colored paste was considered as the reference bait given the results from the analysis of bait preference. Lastly, increases in consumption through time (day = fixed effect) was also tested using methodology above.

## Results

### Bait Preference

Overall, we recorded and processed 249,902 images for the study of bait preference. One camera was stolen on Day 9 and no images were recovered, therefore the total number of study sites was 87. We identified 9,940 images of feral swine (4.0% of images), of which 9,608 contained adult feral swine (Fig 2). There were 13,162 images that contained nontarget species (5.3% of images) from 15 different classifications. White-tailed deer, raccoons (*Procyon lotor*), and birds were the most frequently observed nontarget species (cumulative images = 9,083, or 3.6% of all images).

**Fig 3:**
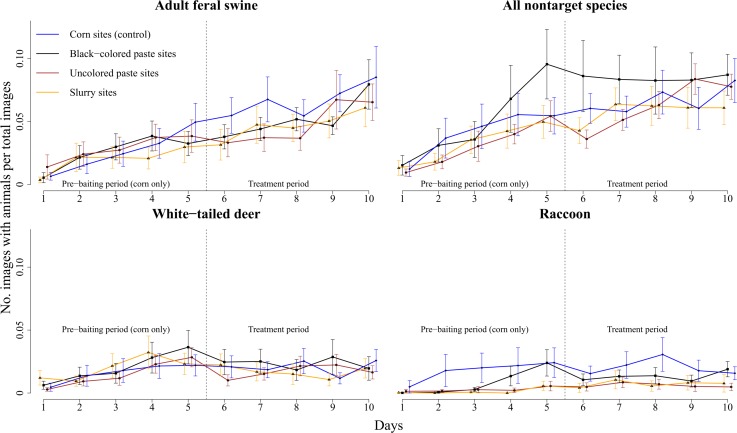
The average rate of visition and standard errors by treatment for 10-day studies at 87 random bait sites for adult feral swine (*Sus scrofa*), all nontarget species combined, white-tailed deer (*Odocoileus virginianus*), and raccoons (*Procyon lotor*) on Camp Bullis and Lackland Air Force Base Medina Annex, TX, USA during April–May 2015.

**Fig 4:**
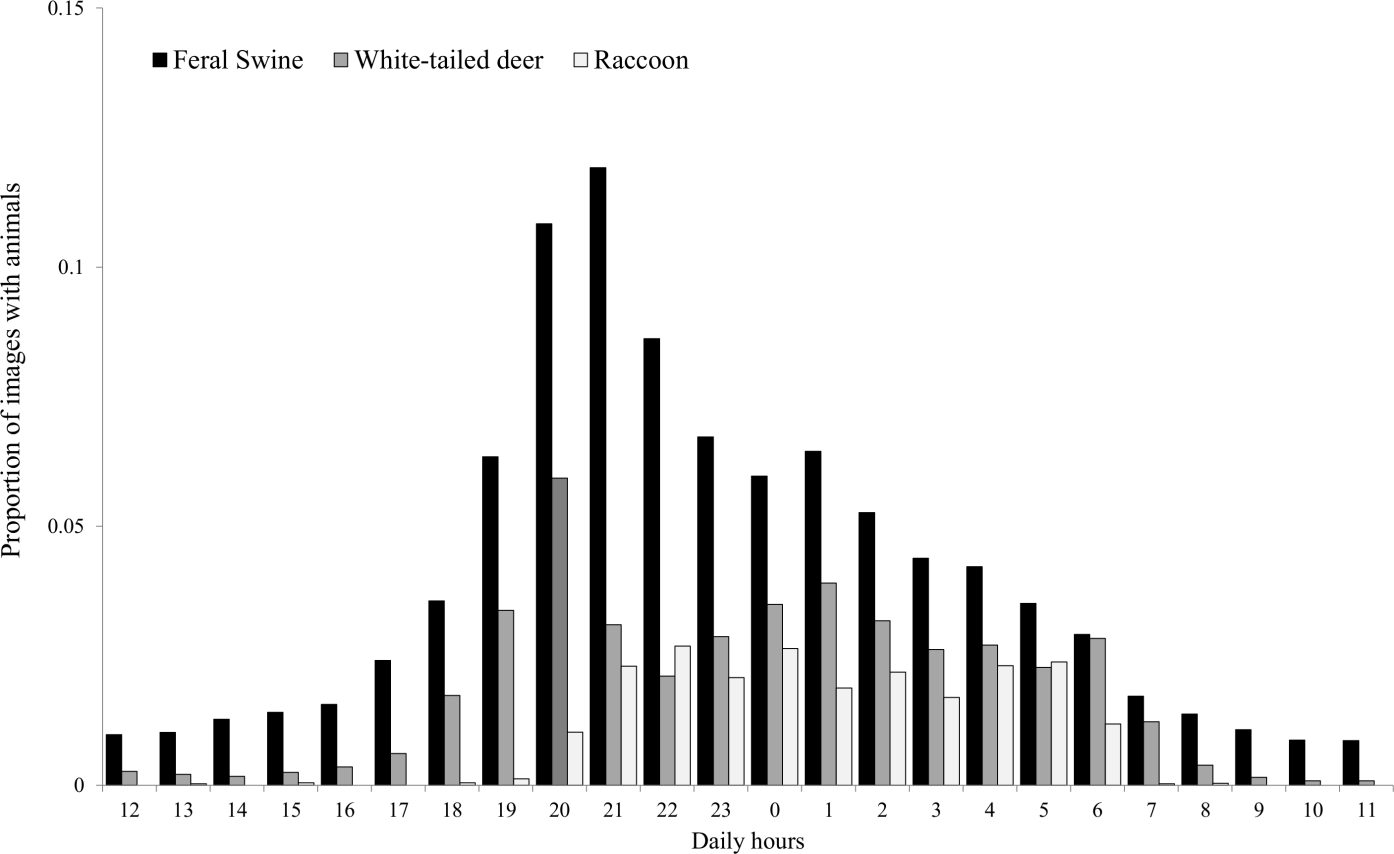
The proportion of average daily times of visitation by feral swine (*Sus scrofa*), white-tailed deer (*Odocoileus virginianus*), and raccoons (*Procyon lotor*) throughout a 10 day study period at 87 random bait sites on Camp Bullis and Lackland Air Force Base Medina Annex, TX, USA during April–May 2015.

We found little evidence that visitation by feral swine differed among sites during the prebaiting period (Table 1), suggesting that none of the preference sites were biased to be in areas with higher or lower densities of feral swine and that populations were equally sampled throughout the study areas. After treatments were deployed during the 5-day treatment period, we observed a decrease in visits to sites with uncolored and slurry pastes, but not to sites with black-colored paste, compared to the control sites. On average, 48 images of feral swine per 1,000 images ($\bar{x}$ = 0.048, SE = 0.014) were observed at sites with uncolored paste, 47 images ($\bar{x}$ = 0.047, SE = 0.013) at sites with slurry, 52 images ($\bar{x}$ = 0.052, SE = 0.007) at sites with black-colored paste, and 67 images ($\bar{x}$ = 0.067, SE = 0.017) at the control sites. However, this difference was not present by the last days (Days 9 and 10) of the study. We found that black-colored paste was visited as much as the control corn during all periods. Overall visitation by feral swine increased throughout the 10-day study period for all treatments (Fig 3). The average duration of visitation by feral swine was ~40 min within the camera field of view. Feral swine showed mostly nocturnal behavior, with a peak in visitation occurring during 1900–0200 hours (Fig 4). The average amount of time taken for the bait to be totally consumed was 658.5 min (SE = 20.4) post-offering, and did not differ among any of the treatment or control sites during the 5 day treatment period. Throughout this period, black-colored paste was totally consumed daily at 65% of sites, whereas 49.5% of sites with uncolored paste, 40.0% of sites with slurry, and 61.8% of control sites had all bait consumed. Black-colored paste had higher average temperatures ($\bar{x}$ = 49.17°C, SE = 2.35) than uncolored paste ($\bar{x}$ = 42.50°C, SE = 1.89) when exposed to sunlight (β = 6.67, 95% CI = 0.58–12.90), however we did not detect a difference in visitations by feral swine between sites with black-colored and uncolored paste during daylight hours (β = 10.30, 95% CI = -8.97–30.20). The largest difference in temperature between the pastes was 10.4°C, recorded at 1300 hrs.

**Table 1:** Parameter estimates and 95% credible intervals for examining preference by feral swine (*Sus scrofa*) amongst 3 treatment bait matrices compared to a control bait (whole-kernel corn) at *n* = 87 random sites on Camp Bullis and Lackland Air Force Base Medina Annex, TX, USA during April–May 2015.

	Count of images		
Treatment	All feral swine	Adult feral swine	Period
Black-colored paste	-0.02 (-0.54–0.50)	-0.00 (-0.53–0.52)	**Prebaiting**
Uncolored paste	0.10 (-0.42–0.60)	0.10 (-0.43–0.59)	**(Days 1–5)**
Slurry	-0.30 (-0.85–0.24)	-0.28 (-0.82–0.26)	
Black-colored paste	-0.21 (-0.53–0.09)	-0.25 (-0.57–0.06)	**5 day treatment**
Uncolored paste	-0.35 (-0.70–-0.03)**	-0.34 (-0.67–-0.003)**	**(Days 5–10)**
Slurry	-0.38 (-0.71–-0.06)**	-0.35 (-0.68–-0.04)**	
Black-colored paste	-0.16 (-0.62–0.31)	-0.24 (-0.72–0.25)	**Last 2 days treatment**
Uncolored paste	-0.21 (-0.68–0.27)	-0.19 (-0.71–0.32)	**(Days 9–10)**
Slurry	-0.38 (-0.88–0.12)	-0.36 (-0.85–0.12)	
Black-colored paste	-0.01 (-0.67–0.67)	-0.05 (-0.75–0.65)	**Last day treatment**
Uncolored paste	-0.26 (-0.96–0.47)	-0.24 (-0.96–0.51)	**(Day 10)**
Slurry	-0.33 (-1.03–0.39)	-0.31 (-1.03–0.43)	

** Indicates evidence of a statistical and biological difference.

White-tailed deer, the most frequent nontarget species, equally visited all the treatments as much as the control corn (Table 2). Considerable variation during the prebaiting period indicated that raccoons and birds had more frequent visitation to some sites, suggesting possible bias in population densities for these animals. However, when all nontarget species were combined, all of the treatments were visited in equal amounts as the control corn, suggesting no preference or avoidance of any treatments. Unlike feral swine, visitation by white-tailed deer and raccoons remained mostly consistent through time for all treatments (Fig 3). All-nontargets combined showed an increasing frequency of visitation throughout the study period for all treatments.

**Table 2:** Parameter estimates and 95% credible intervals for examining preference by common nontarget species; white-tailed deer (*Odocoileus virginianus*), passerine and scavenger birds, raccoons (*Procyon lotor*), and all nontarget species combined, amongst 3 treatment bait matrices compared to a control bait (whole-kernel corn) at *n* = 87 random sites on Camp Bullis and Lackland Air Force Base Medina Annex, TX, USA during April–May 2015.

	Count of images				Period
Treatment	White-tailed deer	Raccoons	Birds	All nontargets	
Black-colored paste	0.26(-0.34–0.87)	-0.76 (-1.72–0.18)	0.47 (-0.14–1.07)	0.25 (-0.20–0.74)	**Prebaiting**
Uncolored paste	-0.08 (-0.54–0.74)	-1.85 (-3.02–-0.72)**	0.43 (-0.22–1.03)	-0.08 (-0.58–0.40)	**(Days 1–5)**
Slurry	0.26 (-0.35–0.90)	-2.65 (-4.07–-1.36)**	-0.88 (-1.59–-0.16)**	-0.19 (-0.66–0.31)	
Black-colored paste	0.12 (-0.44–0.62)	-0.40 (-1.02–0.26)	0.20 (-0.24–0.63)	0.21 (-0.06–0.47)	**5 day treatment**
Uncolored paste	-0.16 (-0.69–0.40)	-1.21 (-1.96–-0.50)**	0.27 (-0.16–0.71)	-0.01 (-0.29–0.27)	**(Days 5–10)**
Slurry	-0.19 (-0.73–0.33)	-1.13 (-1.92–-0.42)**	-0.36 (-0.79–0.10)	-0.14 (-0.41–0.13)	
Black-colored paste	0.20 (-0.68–1.04)	-0.20 (-1.35–0.79)	-0.06 (-0.74–0.59)	0.18 (-0.21–0.60)	**Last 2 days treatment**
Uncolored paste	0.05 (-0.85–0.90)	-1.46 (-2.91–-0.24)**	0.17 (-0.48–0.90)	0.19 (-0.22–0.62)	**(Days 9–10)**
Slurry	-0.22 (-1.08–0.65)	-1.31 (-3.53–0.01)	-0.83 (-1.56–-0.12)**	-0.16 (-0.57–0.27)	
Black-colored paste	-0.29 (-1.45–0.80)	0.11 (-1.15–1.46)	-0.12 (-1.09–0.79)	0.06 (-0.47–0.58)	**Last day treatment**
Uncolored paste	-0.44 (-1.59–0.72)	-1.30 (-3.09–0.32)	0.22 (-0.75–1.15)	-0.04 (-0.57–0.48)	**(Day 10)**
Slurry	-0.28 (-1.41–0.89)	-0.97 (-3.03–0.59)	-1.18 (-2.26–-0.12)**	-0.30 (-0.84–0.24)	

** Indicates evidence of a statistical and biological difference.

### Bait Consumption

**Fig 5:**
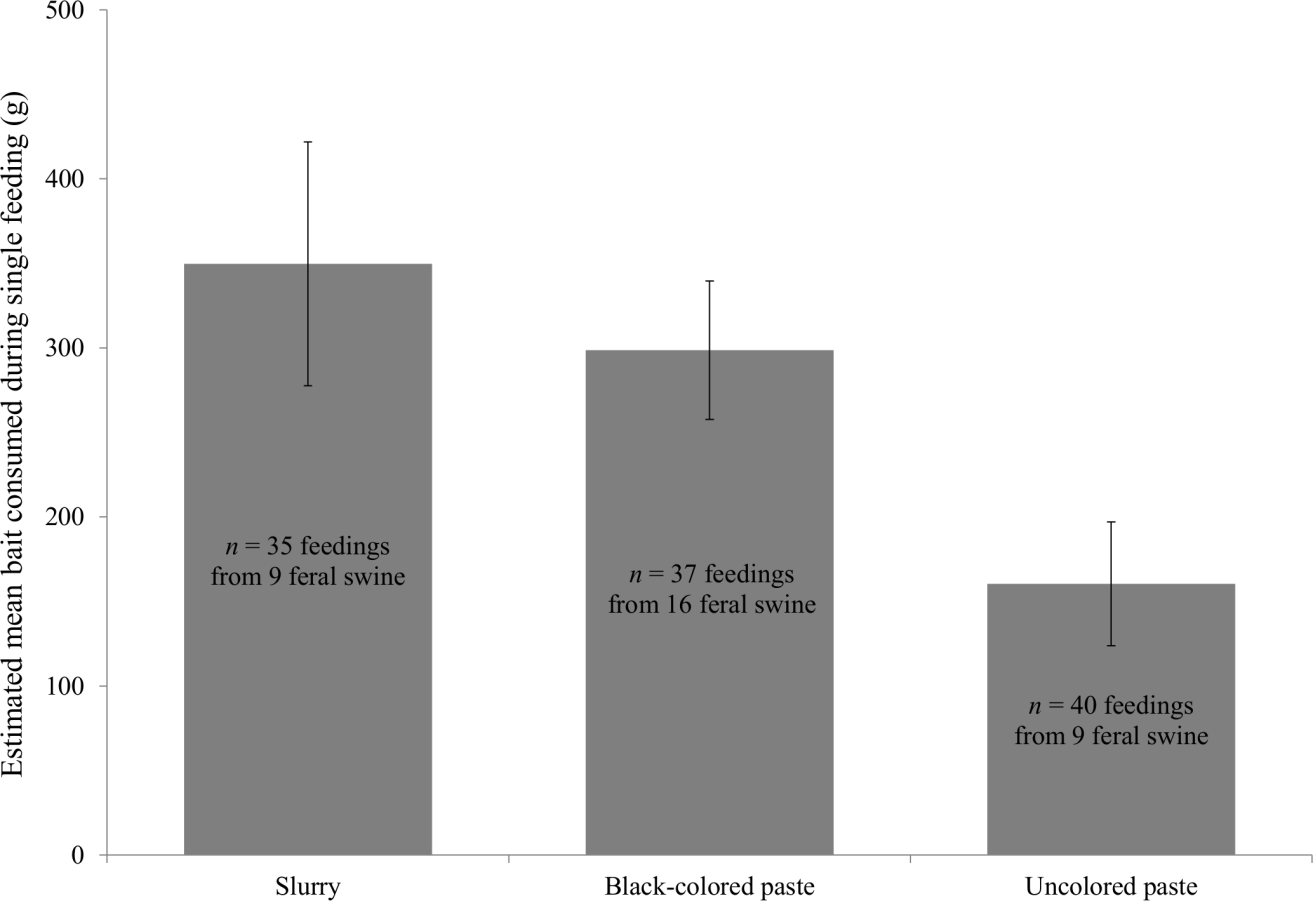
The mean estimated weight (g) and standard errors of 3 treatment bait matrices consumed per feeding event by individual feral swine at 5 sites on Camp Bullis, TX, USA during April 2015.

Overall, 36,506 images for the study of bait consumption were collected, of which 51 individual feral swine were identified. Nineteen of these were recorded feeding for a total of 112 feeding events. The other 32 individuals were not observed feeding because the baits were already consumed, possible shyness to grid boards, or other feral swine were occupying or dominating the grid boards. The average number of feeding events recorded per day for feral swine was 2.2 (range = 1–14 feedings). The average length of a feeding bout was 14.2 min (range = 0.1–136.6 min) and the average length of time between feeding bouts was 29.1 min (range = 2.4–196.0 min) when bait was available. On average, uncolored paste ($\bar{x}$ = 160.5 g, SE = 36.6) was consumed less than black-colored paste ($\bar{x}$ = 298.6 g, SE = 41.0; β = -0.67, 95% CI = -1.21–-0.14; Fig 5). The slurry ($\bar{x}$ = 349.6 g, SE = 72.1) and black-colored paste were consumed in similar amounts (β = -0.14, 95% CI = -0.59–0.34). Of the feral swine that were recorded feeding, we found no evidence that the presence of other feral swine influenced the amounts of bait consumed by individual animals (β = -0.04, 95% CI = -0.60–0.60). Also, we did not find evidence that feral swine consumed more bait through time (β = 0.06, 95% CI = -0.04–0.17).

## Discussion

We identified reduced visitations to the uncolored peanut paste and the peanut slurry matrices, relative to whole-kernel corn. However, this reduction was not detectable by the final days of the treatment period, suggesting that feral swine needed time to acclimate to these treatments. No reduction in visitation was detected for the black-colored peanut paste compared to the control, and this treatment was consumed more often than all other treatment matrices, and consumed similarly to whole-kernel corn (control). Therefore, we surmise that black-colored peanut paste is the most effective option for delivering micro-encapsulated sodium nitrite to feral swine, assuming this matrix will maintain the integrity of micro-encapsulated sodium nitrite through time.

The only difference between the black-colored and uncolored pastes was a small amount of colorant, which caused the black-colored paste to absorb more heat when exposed to sunlight. However, we found no evidence that this warmer paste was more attractive to feral swine, because visitations to both pastes were equal during daylight hours with sun exposure. Therefore, feral swine may have exhibited less visual aversion to the darker colored paste, possibly because the dark appearance reduced neophobic responses. A possible explanation is that the black colorant made the paste appear similar to soil that would be encountered during normal rooting behaviors (i.e., feral swine overturn soil and vegetation to find food) [3, 39, 40]. Similarly, the tan-brown color of uncolored paste and slurry may have appeared more novel to feral swine. Some reports have also indicated that swine can discriminate among colors [41, 42], but behavioral responses to black or any other colors are not well documented [43].

None of the treatments appeared to reduce visitation by nontarget species, which is not surprising given the degree of overlap between food items of feral swine and other wildlife [9, 44]. However, peanut paste may represent an improvement over previously tested baits as feral swine were the dominant species that visited the bait sites (i.e., 43% of images with animals), compared with 22% for a grain-based bait containing fish flavor (PIGOUT^®^), and 51% consumed by raccoons and 20% by collared peccaries (*Pecari tajacu*) in Texas [19]. We also found that visitation by the most common nontarget species, white-tailed deer and raccoons, did not increase after the treatments were offered, suggesting that the treatment matrices were not so desirable as food items to increasingly influence feeding behavior for those species. Lastly, we found no evidence that the black colorant reduced nontarget visitation as hoped. However, the dark appearance may have application for reducing accidental consumption by humans, particularly children.

Results from the consumption of baits using grid boards support the conclusion that feral swine preferred black-colored paste. Black-colored paste was consumed more by individual pigs than uncolored paste. In addition, compared to the slurry, black-colored paste will be able to contain higher concentrations of micro-encapsulated sodium nitrite per volume because it will not have to be diluted with a carrier (e.g., whole-kernel corn). Based on our findings, the average amount of bait consumed during a single feeding (~300 g) was enough to rapidly deliver a lethal dose of sodium nitrite (i.e., ~400 mg/kg) [22] to feral swine weighing ≤71 kg, with a 10% concentration of sodium nitrite in paste. However, lower doses of sodium nitrite (≥135mg/kg) have also been lethal for feral swine [21, 22]. Adult feral swine in the US average 70–100 kg depending on sex [2], indicating that the average consumption in this study could have delivered lethal doses of sodium nitrite to most adult feral swine in 1–2 feeding events. An average of 29 min between feedings likely is short enough for feral swine to consume a lethal dose of sodium nitrite that is micro-encapsulated and inside a bait, before the onset of symptoms [22]. However, we expect that consumption during a feeding bout could be higher than ~300 g without the use of grid boards, because some feral swine appeared unwilling to eat from the boards.

The present results provide information on improved strategies for baiting feral swine to ensure safe and effective delivery of micro-encapsulated sodium nitrite. Due to visitation and consumption by nontarget species, feral swine-specific bait delivery device will be necessary to reduce direct secondary hazards, as discussed in other studies [18, 45–48]. Most feral swine considered as young of year were observed when adult feral swine were present, thus the delivery device should include simultaneous feeding opportunity for young and adults. The most common nontarget species, white-tailed deer and raccoons, visited the baits during similar hours as feral swine, thus timed delivery will not provide an option for reducing nontarget hazards. However, pre-baiting in this study was shown to increase feral swine visitation through time, compared to nontargets, and this greater influence on feeding behavior may be used to specifically target feral swine. Similar findings from previous studies also demonstrate the importance of pre-baiting periods to maximize delivery of a toxicant [18, 47]. These studies suggested anywhere from 6–36 days of pre-baiting were needed to maximize visitation by feral swine, which is supported by the continually increasing visitation we observed throughout a 10 day period. Lastly, more than enough bait needs to be provided so that feral swine have the opportunity to consume lethal doses in short duration.

## Conclusions

Of the 3 bait matrices tested, black-colored paste was the only matrix that did not produce a neophobic response from feral swine when initially introduced. Feral swine exhibited similar preference for this paste as highly-desirable whole-kernel corn, suggesting that this bait is an effective matrix for delivering micro-encapsulated sodium nitrite to feral swine. They consumed enough of the black-colored paste to ingest lethal doses of sodium nitrite in 1–2 feedings, confirming the utility of this matrix. None of the matrices minimized visitations by nontarget species, so we recommend use of feral swine-specific bait stations to prevent non-target access to toxic baits in the United States. Several additional studies and advancements will be required prior to registration of micro-encapsulated sodium nitrite as a toxicant for feral swine, and we are steadfastly working toward this.
